# Efficacy of Bilateral Mental Nerve Block with Bupivacaine for Postoperative Pain Control in Mandibular Parasymphysis Fractures

**DOI:** 10.5681/joddd.2014.031

**Published:** 2014-09-17

**Authors:** Ali Hossein Mesgarzadeh, Hosein Afsari, Sohrab Pourkhamne, Mohamadreza Shahamfar

**Affiliations:** ^1^Associate Professor, Department of Oral and MaxilloFacial Surgery, Faculty of Dentistry, Tabriz University of Medical Sciences, Tabriz, Iran; ^2^Oral and Maxillofacial Surgeon, Private Practice, Tehran, Iran; ^3^Assistant Professor, Department of Orthodontics, Faculty of Dentistry, Ardebil University of Medical Sciences, Ardebil, Iran; ^4^Post-graduate Student, Department of Orthodontics, Faculty of Dentistry, Tabriz University of Medical Sciences, Tabriz, Iran

**Keywords:** Bupivacaine, mandibular fracture, pain

## Abstract

***Background and aims.*** Postoperative pain control is extremely important for both patients and surgeons; in this context, long-acting local anesthesia can play an important role after open reduction of maxillofacial fractures. The purpose of this study was to evaluate the effect of bilateral mental nerve block with bupivacaine on postoperative pain control in mandibular symphyseal fractures.

***Materials and methods.*** Fifty patients with pure mandibular symphyseal fractures were studied in two control and study groups. In contrast to the control group, the study group received bilateral mental nerve block with bupivacaine postoperatively. Patients were examined in relation to pain severity and opioid analgesic drug need sequences.

***Results.*** The study group needed significantly less opioid than the control group (P<0.01, U=141). The control and study groups were different in first opioid administration time. The control and study groups received first opioid dose in 0-2 and 2-4 hours, respectively.

***Conclusion.*** Bilateral mental nerve blocks with bupivacaine can reduce opioid analgesic need and it has a positive effect on postoperative pain control in mandibular symphyseal fractures.

## Introduction

Postoperative pain control is one of the most important phases of most surgical procedures. Due to the surgical trauma and subsequent cascade release of pain mediators, the receptors of pain are stimulated, resulting in various degrees of pain in the postoperative period. Therefore, both the surgeon and anesthesiologist are involved and interested in pain control during and after the operation. Various methods of analgesic drug administration have been proposed by clinicians to help relieve patients’ pain experience immediately after surgery. The goal of every postoperative pain management technique is to reduce or eliminate pain and discomfort with minimum side effects. Opioid drug administration is a common technique to reduce pain from surgical trauma. However, the use of large doses of opioid drugs during and after surgery can be associated with an increased incidence of ventilatory depression, sedation, nausea, vomiting, pruritus, difficult voiding and ileus.^1^ In maxillofacial surgeries in which patients often receive intermaxillary fixation, these side effects are at best estimate, a nuisance to both the patient and surgeon and at worst estimate a life-threatening complication. Ventilatory depression and vomiting are among the latter category, especially in early postoperative hours. Various methods have been proposed to minimize these side effects. Nerve block with long-acting local anesthesia is a proposed technique in this regard. These solutions have been used in many procedures like limb fractures with open reduction, graft donor site surgeries, laparoscopy and arthroscopy. Femoral nerve block was used for extracapsular femoral neck fracture by Haddad and Williams in 1995.^2^ Dashow, Coulthard, Hoard, Sbitany, Haenel and Furst studied the effect of bupivacaine as a local anesthetic for pain control and morbidity reduction of iliac bone harvesting sites, rib fractures and temporomandibular joint arthroscopy.^3-6^ In maxillofacial field, bupivacaine has been used as a safe local anesthetic solution for pain control after cleft lip operation or third molar surgery, alone or in combination with low-power laser and diclofeanc.^7-13^ Bupivacaine is a well-known long-acting local anesthesia solution.^14^ The aim of the current study was to evaluate the efficacy of bi-lateral mental nerve block with bupivacaine in post-operative pain control and reduction of total analgesic drug need in patients referring to a trauma center with mandibular sysmphyseal fractures.

## Materials and Methods

A double-blind case-control prospective study was performed on the patients with pure parasymphyseal fractures, referred to the Oral and Maxillofacial Department Clinic for treatment of their fractures. Patients were examined clinically by clinical staff. Their pure parasymphyseal fractures were confirmed by proper radiographic examinations. Before initiation of the study, its protocol was approved by institutional ethics committee. All the patients and their relatives were informed about the details of the study and written informed consent was obtained by the researchers. Sample size was estimated by using data of a previous pilot study. A total of 50 patients were randomly allocated to two groups (25 patients in the study group, 25 patients in the control group) using a random number generator. Inclusion criteria consisted of patients having pure symphyseal fracture, who did not have any systemic diseases and did not use any medication that would alter pain perception. All the patients with any history of allergy to local anesthetics and those under 18 were excluded. Anesthesia protocol was planned by an anesthesiologist with the same drugs for all the patients: 2 mg/kg propofol, 2 μg/kg fentanile, 0.05 mg/kg midazolam, 0.4 mg/kg for induction of anesthesia, %50 N_2_O, %5 O_2_, isoflurane 1-1.5% for maintenance and 1.5 mg atropine and 2,5 mg neostigmine for reversing of the anesthesia.

**Figure 1.**
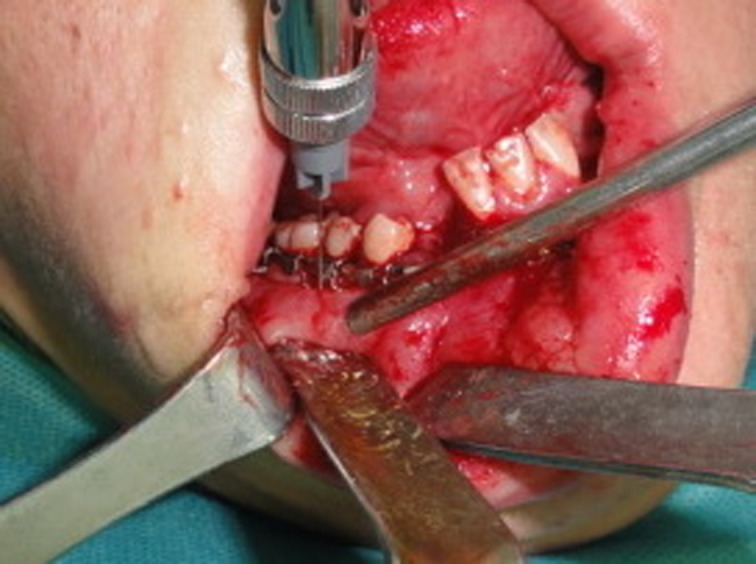
Mental nerve block technique.

Both the patients and surgeon were blinded to the use of local anesthesia since the labels of anesthetic solution were pulled off and the cartridges were coded by one of the nursing staff who was not directly involved in data collection. The same surgeon performed all the surgeries using an intraoral approach according to the Champys’ miniplate fixation rules. Mental nerve is usually located between the apices of the first and second premolars. The mucobuccal fold and mandibular premolars are good landmarks for this block. In the study group at the end of surgery, immediately after completion of surgical wound closure, two mL of 0.5% bupivacaine hydrochloride with 1:200000 epinephrine were injected for each side (Sinobright Pharmaceutical Co., Beijing, China) using a 25-gauge short needle toward the apices of mandibular premolars with bevel orientation toward the bone. The depth of penetration was 5 to 6 millimeter without entering into the mental foramen. Then the syringe was aspirated and if negative, anesthetic solution was injected over 20 second (Figure 1).

The control group did not receive any nerve block injection. Pain severity was assessed by using visual analog scale from 0 to 10. Pain assessment was performed from the time of extubation and in the recovery room up to 24 hours by nursing staff that were blinded to the intervention. If the pain score was over 6, the patients (regardless of the study or control group) received 25 mg of pethidine intramuscularly (pethidine hydrochloride Gerot Pharmazeutik, Vienna, Austria) for their postoperative pain as a backup analgesic drug according to regular nursing observation and hospital protocol. All the patients were examined in relation to pain severity and opioid analgesic need sequences. Mann-Whitney U test was used to compare the opioid analgesic drug need and first opioid dose time. Data were analyzed with SPSS 16 and statistical significance was set at P<0.05.

## Results

Forty male and 10 female subjects, with an age range of 18-40 years, met all the criteria for this research. The patients’ average body weight was 64 kg ranging from 53 kg to 77 kg. No significant differences were found between age and gender (P>0.05) in both groups. There was a significant total mean difference between the intervention and control groups in the frequency of opioid drug need the study group needed less opioid than the control group (P<0.001, U=141) (Table 1). Seven patients (28%) in the intervention group did not exhibit any need for analgesic drugs but all the control group patients needed opioid drugs postoperatively (Table 1 ). Twenty-two (88%) patients from the control group received first analgesic drug within 0-2 hours postoperatively while only one patient from the study group required opioid drug injection during the same period of time. There was a significant difference between the study and control groups in the first analgesic injection requirement (P<0.01, U=38.5) (Table 2 ). Moreover, the two groups showed significant differences in the second and third times of analgesic requirements (P=0.002).

**Table 1:** Number of patients with analgesic drug need 24 hours postoperatively in the studied groups

Groups	Frequency of analgesic drug need			
	0	1	2	3
Control group	0	8	15	2
Study group	7	12	6	0

**Table 2:** Number of patients receiving first postoperative analgesic drug injection in the studied groups

Groups	Hours after complete consciousness			
	0-2h	2-4h	4-6h	<6h
Control group	22	3	0	0
Study group	1	17	0	0

## Discussion

Due to the severity of pain and other complications, early postoperative hours are very important for both the patients and surgeons. Opioid administration is a common way to overcome the pain sequelae in nearly all the surgeries including those in the maxillofacial region. However, the vomiting side effect associated with the opioids may be a especially life-threatening complication in maxillofacial surgeries since these patients often undergo intermaxillary fixation which inhibits the proper drainage of the vomit from the oral route, adding an increased risk of pulmonary aspiration. Therefore, reduction of the opiod dose needed after surgery may be a prudent clinical objective after maxillofacial surgeries. Long-acting anesthesia on the other hand may provide a pain relief gate for the patient, reducing the need for opioids. These anesthetics have been examined in various surgical procedures. Bouloux and Punnia-Moorthy in a randomized, double-blind, crossover study compared the clinical use of bupivacaine and lidocaine in third molar surgery in two separate sessions. Bupivacaine was used for the third molar surgery on one side, whereas lidocaine was used for the other side. Pain experience, analgesic consumption, cardiovascular response, blood concentration and systemic toxicity were evaluated. Bupivacaine significantly reduced postoperative experience only for eight hours. The results of their study did not show a difference in a variety of parameters, other than postoperative pain experience.^15^ Hyrkas et al^16^ studied the use of bupivacaine and lidocaine in combination with sustained-released preparation of diclofenac in postoperative pain control of third molar surgery. They reported high efficiency of combined bupivacaine versus lidocaine. The same results were achieved by Singh and Bhardwaj^17^ using continuous mandibular nerve block with bupivacaine for postoperative pain relief of mandibular fracture surgeries. The present study confirms previous investigations. Generally, opioid drugs are a common way of pain control in nearly all the surgeries. The important things that we should take into account are the side effects of drugs. In some instances side effects are the unavoidable part of drugs. Perrott et al^18^ found postoperative nausea and vomiting to be the most common postoperative complications after oral and maxillofacial surgeries. Silva et al^19^ reported a prevalence rate of 40% for these complications after maxillofacial surgeries. Bilateral mental nerve block can reduce the need for opioid analgesic drugs, eliminating the drugs’ side effects. This method can also be a useful technique for combined maxillofacial and brain traumatic patients. Ventilatory depression and sedation, on the other hand, can also be fatal for these patients. Significant time difference for the first needed dose of opioid in the study group provides a good opportunity for surgeons to evaluate the level of patients’ consciousness. Vomiting and aspiration risk can be doubled in emergency situation and urgent maxillofacial surgeries. Bilateral symphyseal fracture in unconscious patients is an emergency situation because posterior displacement of fractured symphysis by genioglossus and geniohyoid muscles can block the airway concomitant with tongue position. Use of bilateral mental nerve block at the end of the surgery delays the first opioid drugs need and reduces the total dose of analgesic drugs; therefore, medical and nursing staff have enough time to re-evaluate the level of patients’ medical and consciousness conditions without any sedative drug interaction. Addiction to opioid analgesics is yet under clinical and psychosocial investigation. Risk of addiction in psychologically susceptible patients is another side effect of opioid drugs. Reduction of total post-operative opioid doses and patients’ need may have an effective influence on such issues.

## Conclusion

For reasons mentioned above, bilateral mental nerve block with bupivacaine can be an effective and safe method for reducing opioid  drugs need and controlling of postoperative pain and discomfort in mandibular symphyseal fractures.
